# Abstract of the Transactions of the American Medical Association

**Published:** 1864-07

**Authors:** 


### MISCELLANEOUS.

### ABSTRACT OF THE TRANSACTIONS OF THE AMERICAN MEDICAL ASSOCIATION.

### *FIFTEENTH ANNUAL MEETING.*

Condensed from the "New York Medical Independent."

[CONCLUDED.]

THIRD DAY—MORNING SESSION.

The Convention re-assembled this morning.

Dr. MAURAN, of Rhode Island, called the attention of the Convention to the prizes offered by Rhode Island, amounting to $200, for the best essay on the following thesis:

1—What evidence is there that inflammatory and febrile diseases have undergone any general change of type?

2d—The effects of climate in America on Tuberculous diseases.

Dr. E. R. SQUIBB of New York, from Committee on the Practical Workings of the United States Law relating to the Inspection of Drugs and Medicines, presented a report.

Dr. STORER read the following resolutions, which were adopted:

*Resolved*, That in the opinion of the American Medical Association, it is expedient that there should be attached to every public hospital for the insane, one or more consulting physicians, who may be consulted at the discretion of the Superintendent; such measure being for the interest of the hospital, its medical officers, and its patients.

*Resolved*, That a copy of the above resolution be transmitted to the Board of Trustees of each of our public hospitals for the insane, and also to the Secretary of the Association of American Superintendents, with the request that it may be endorsed by that body, and the act proposed be urged upon the respective boards with which the members are officially connected.

The Committee on Nominations, Dr. ARMOUR of Michigan, reported the following Standing Committees:

On Insanity—Ralph Hills, Ohio; C. H. Nichols, District of Columbia; D. P. Bissell, N. Y.; S. W. Butler, Pa.; John S. Butler, Ct.; E. R. Van Duser, Mich.

On Exsection and its Connection with Conservative Surgery—Lewis A. Sayre, N. Y.; G. W. Norris, Pa.; G. C. Blackman, Ohio; H. S. Tewksberry, Me.; E. Andrews, Ill.; G. B. Twitchell, N. H.; G. C. Hughes, Iowa; George Clymer, U. S. N. J. R. W. Dunbar, Md.; R. H. Gilbert, U. S. A.

On lcohol and its Relation to Man—G. E. Morgan, Md.

On Microscopic Observations on Cancer Cells—L. J. Sanford, Ct.

On Medical Ethics—J. J. Murphy, Ohio; M. L. Linton, Mo.; B. F. Schenck, Pa.; S. Wickersham, Ill.; A. J. Fuller, Me.

On the Microscope—J. M. Corse, Pa.

On Quarantine—D. D. Clark, Pa.; E. M. Snow, R. I.; W. Jewell, Pa.; E. D. Fenner, La.; J. W. Houck, Md.

On Causes of the Extinction of the Aboriginal Races of America—G. Suckley, New York.

On International Medical Ethics—Robert Thompson, Ohio; G. Shattuck, J. B. Upham, Mass.; G. C E. Weber, Ohio.

On the Relations which Electricity sustains to the Cause of Disease—S. Littell, Pa;

On the Morbid and Therapeutic Effect of Mental and Moral Influences—A. B. Palmer, Mich.

On the Causes and Treatment of Ununited Fractures—F. H. Hamilton, N. Y.

On Diphtheria—L. Clark, Ill.

On the Drainage and Sewerage of large Cities, and their Influences on Public Health—W. J. C. Dehamil, Dist. of Col.; E. C. Baldwin, Md.; C. Ramsay, N. Y.

On the Uses and Abuses of Pessaries—J. P. White, N. Y.

On International Medical Ethics, to Investigate the Conditions demanded for a Diploma of Doctor of Medicine in the various Medical Schools and Universities of Europe—J. B. Upham, Mass.; R. Thompson, Ohio; G. C. Shattuck, Mass.; G. C. E Weber, Ohio.

On Climatology and Epidemic Diseases—C. W. Parsons, R. I.; P. A. Stackpole, N. H.; T. M. Logan, Cal.; J. R. C. Hamill, Ill.; J. C. Weston, Me.; B. H. Catlin, Ct.; C. L. Allen, Vt.; T. Antisell, Dist. of Col.; J. W. H. Baker, Iowa; A. Sager, Mich.; O. S. Mahon, Md.; J. W. Russell, Ohio; D. F. Condie, Pa.; H. Townsend, New York.

On Autopsies in relation to Medical Jurisprudence—F. C. Finnell.

On so-called Spotted Fever—J. J. Levick.

On the Introduction of Disease by Commerce, and the means for its Prevention—A. N. Bell, N. Y.

On Patent Rights by Medical Men—D. Prince, Ill.; T. Antisell, Dist. of Col.; S. Smith, N. Y.

Dr. W. B. Atkinson of Philadelphia, was proposed for Permanent Secretary of the Association, and Dr. Storer of Boston, for Assistant Secretary.

Dr. Kennedy moved the substitution of the name of Dr. Furman as Secretary, paying a high eulogium to the character of Dr. Furman.

The nomination of Dr. Furman was lost by a vote of 36 for 75 against, whereupon Dr. W. B. Atkinson was elected unanimously.

The Committee on Prize Essays, through Dr. Peaslee of New York, reported that they had received three essays for competition, viz: On the Surgical Treatment of Morbid Growths in the Larynx, which was received too late to be acted upon. This essay was written with excellent care, and showed great research on the subject.

What effect has the milk and meat of diseased animals on public health?

The Committee gave this a high eulogium and expressed a hope for its publication.

The third essay, "Pathology of Jaundice," was given the prize. Dr. J. Fleet Spier of Brooklyn, N. Y.

The meeting then adjourned to 4 o'clock P. M.

AFTERNOON SESSION.

The meeting was called to order by the President, and the roll of delegates called by the Secretary.

A resolution was passed, amending the by-laws so as to elect the President by ballot.

A resolution of thanks to the profession of this city for their hospitality, was passed.

Also, a resolution recognizing the services, patriotism and sacrifices of the army surgeons.

Also, a vote of thanks to Secretary Furman.

Also, a resolution requesting Rail Road Companies to convey delegates at half fare.

Also, a resolution to appoint a Committee to codify the ordinances of the Association.

A resolution on the death of Dr. B. F. Bache of Philadelphia, was referred to the Committee on Necrology.

Report of Section on Chemistry was adopted.

Dr. J. Homberger offered the following:

*Whereas*, The position of Specialists and Specialties is but very ill-defined, be it

*Resolved*, That the American Medical Association go into a Committee of the Whole, in order to define the position of Specialism and Specialists, as well as the duties of the profession towards Specialists, and the duties of Specialists.

This resolution was followed by an animated debate. It was referred to a Committee of five, of which Dr. Homberger is Chairman.

Resolution to memorialize the Legislature to enact a law providing for the compensation of medical men when attending as witnesses in courts.

Report of Committee on Epidemics, etc., was adopted.

A resolution to apprise Congress of the unworthiness of Dr. Morton for a reward as the inventor of Sulphuric Ether as an anæsthetic, was passed.

Report of Section on Surgery was adopted.

The amendment to the Constitution providing for the taking of the

Chair by the President elect at the succeeding meeting, was also adopted.

A motion to offer a prize for an Essay on Abortion, was referred to the Committee on Prize Essays.

A vote of thanks to President DAVIS for the able manner in which he presided over the meeting, was unanimously passed.

After which the Association adjourned to meet in Boston on the first Tuesday in June, 1865.

